# Prolonged Targeted Cardiovascular Epidural Stimulation Improves Immunological Molecular Profile: A Case Report in Chronic Severe Spinal Cord Injury

**DOI:** 10.3389/fnsys.2020.571011

**Published:** 2020-10-15

**Authors:** Ona Bloom, Jill M. Wecht, Bonnie E. Legg Ditterline, Siqi Wang, Alexander V. Ovechkin, Claudia A. Angeli, Anthony A. Arcese, Susan J. Harkema

**Affiliations:** ^1^VA RR&D National Center for the Medical Consequences of Spinal Cord Injury, James J. Peters VA Medical Center, Bronx, NY, United States; ^2^Institute of Molecular Medicine, The Feinstein Institutes for Medical Research, Manhasset, NY, United States; ^3^Departments of Molecular Medicine; Physical Medicine and Rehabilitation, Donald and Barbara Zucker School of Medicine at Hofstra-Northwell, Hempstead, NY, United States; ^4^Department of Medicine, The Icahn School of Medicine, Mount Sinai, New York, NY, United States; ^5^Rehabilitation Medicine, The Icahn School of Medicine, Mount Sinai, New York, NY, United States; ^6^Kentucky Spinal Cord Injury Research Center, University of Louisville, Louisville, KY, United States; ^7^Department of Neurosurgery, School of Medicine, University of Louisville, Louisville, KY, United States; ^8^Department of Bioengineering, University of Louisville, Louisville, KY, United States

**Keywords:** spinal cord injury, epidural stimulation, neuromodulation, immune system, orthostatic hypotension, blood pressure regulation, cerebral blood flow velocity, cardiovascular regulation

## Abstract

In individuals with severe spinal cord injury (SCI), the autonomic nervous system (ANS) is affected leading to cardiovascular deficits, which include significant blood pressure instability, with the prevalence of systemic hypotension and orthostatic intolerance resulting in an increased risk of stroke. Additionally, persons with SCI rostral to thoracic vertebral level 5 (T5), where sympathetic nervous system fibers exit the spinal cord and innervate the immune system, have clinically significant systemic inflammation and increased infection risk. Our recent studies show that lumbosacral spinal cord epidural stimulation (scES), applied at the lumbosacral level using targeted configurations that promote cardiovascular stability (CV-scES), can safely and effectively normalize blood pressure in persons with chronic SCI. Herein we present a case report in a female (age 27 years) with chronic clinically motor complete cervical SCI demonstrating that 97-sessions of CV-scES, which increased systemic blood pressure, improved orthostatic tolerance in association with increased cerebral blood flow velocity in the middle cerebral artery, also promoted positive immunological changes in whole-blood gene expression. Specifically, there was evidence of the down-regulation of inflammatory pathways and the up-regulation of adaptative immune pathways. The findings of this case report suggest that the autonomic effects of epidural stimulation, targeted to promote cardiovascular homeostasis, also improves immune system function, which has a significant benefit to long-term cardiovascular and immunologic health in individuals with long-standing SCI.

**Clinical Trial Registration:** www.ClinicalTrials.gov, identifier NCT02307565.

## Introduction

Severe cervical spinal cord injury (SCI) results in multi-organ system dysfunction that stems, in part, from impaired descending supraspinal control of the autonomic nervous system (ANS). As a result of ANS impairment, cardiovascular dysfunction is common and takes the form of blood pressure instability, which includes persistent hypotension, orthostatic hypotension, and autonomic dysreflexia. These blood pressure disorders have been implicated in an increased prevalence of cerebrovascular compromise (Saleem et al., 2018), cognitive deficits (Wecht et al., 2018), and stroke (Wu et al., 2012), which are independent of symptom reporting in the majority of individuals with SCI. It is thought that an additional consequence of ANS impairment is immune system dysfunction (Schwab et al., 2014; Herman and Bloom, 2018), which promotes systemic inflammation, and may also play a role in long-term cardiovascular morbidity and mortality following SCI.

The maladaptive reorganization of the ANS following SCI is independent of the level of injury as assessed by the International Standards for the Neurological Classification of SCI (ISNCSCI; Gimovsky et al., 1985; Currie and Krassioukov, 2015; Katzelnick et al., 2019). Although blood pressure instability appears to be more prevalent in individuals with cervical SCI (Claydon and Krassioukov, 2006; Wecht et al., 2013), it is not exclusive to this group because a critical proportion of thoracic sympathetic neurons integral to facilitating global vasomotor control are decentralized by spinal lesions occurring at or above thoracic vertebral level 1 (T1). Additionally, similar to astronauts returning from space flight (Convertino, 2009; Hargens and Richardson, 2009), blood pressure instability in individuals with SCI at any level may stem from reduced orthostatic pressure gradients due to the limited amount of time chronic wheelchair users spend in an upright standing position (Vaziri, 2003). Importantly, although most individuals with chronic cervical SCI do not report symptoms associated with cerebral hypoperfusion, mounting evidence suggests that asymptomatic daily fluctuations in blood pressure along with persistent hypotension are associated with reduced cerebral blood flow velocity in the middle cerebral artery (Phillips et al., 2014a,b, 2018; Wecht et al., 2017), and cognitive impairments (Jegede et al., 2010; Phillips et al., 2014b; Wecht et al., 2018). Individuals with SCI-induced blood pressure instability report that this condition restricts their participation in daily activities, and diminishes their independence, vitality, and quality of life (Weaver et al., 2007; Carlozzi et al., 2013).

After SCI, ANS impairment promotes immune system dysfunction *via* diminished descending supraspinal control and by damage to the sympathetic pre-ganglionic neurons that innervate immune organs at or below the thoracic level 5 (T5; Schwab et al., 2014; Herman and Bloom, 2018), which also corresponds to sympathetic neuronal innervation of cardiovascular targets. Two clinically relevant aspects of immune dysfunction after SCI are systemic inflammation and increased risk of infection. Chronic systemic inflammation, which promotes the risk of cardiovascular disease and stroke in all populations, is present in more than 75% of individuals living with SCI (Nash et al., 2016; Bloom et al., 2020) and is highest in individuals with the greatest neurological dysfunction and the least mobility (Morse et al., 2008; Bloom et al., 2020). Individuals with the greatest neurological dysfunction have the greatest risk for infection, and recent studies have demonstrated that the number of infections during the first year after injury correlates inversely with neurological recovery in individuals with cervical SCI (Failli et al., 2012; Brommer et al., 2016; Nash et al., 2016; Bloom et al., 2020). To better understand the molecular mechanisms contributing to increased systemic inflammation and infection risk after SCI, we recently analyzed gene expression changes in whole blood from individuals with chronic (>12-months post-injury) SCI and discovered broad upregulation of pro-inflammatory genes, in particular the Toll-like Receptor (TLR) pathway, a key signaling pathway for pathogen recognition and subsequent activation of innate immunity (Herman and Bloom, 2018; Herman et al., 2018). Also, we discovered the decreased expression of genes from natural killer (NK) cells, which are well-understood to be critical for fighting viral infections. Both of these molecular observations are significantly pronounced in individuals with SCI rostral to T5.

Recent findings that describe the use of targeted lumbosacral epidural stimulation to restore cardiovascular homeostasis (CV-scES) in individuals with chronic SCI indicate blood pressure can be immediately stabilized within a normotensive range for prolonged periods. Moreover, even when tested without active stimulation, the improved blood pressure regulation persists, and orthostatic hypotension is alleviated after 80-sessions of 2-h per day stimulation in a home setting (Harkema et al., 2018). Active epidural stimulation in the T11-L1 region in an individual with chronic C5, motor complete SCI has been reported to increase cerebral blood flow velocity during head-up tilt (West et al., 2018); however, the effects of a prolonged CV-scES intervention on cerebral blood flow velocity during a head-up tilt maneuver have not been reported. We explored here the effects of a prolonged CV-scES intervention on blood pressure and cerebral blood flow regulation and hypothesized that improved ANS function may also positively impact the immune system. The goals of this case study were to determine the effects of targeted CV-scES on orthostatic blood pressure and cerebral blood flow velocity responses during a head-up tilt maneuver and associated changes in whole blood gene expression in an individual with severe, chronic, cervical SCI.

## Materials and Methods

**Figure 1:**
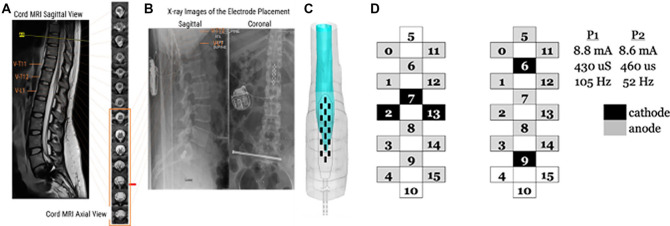
Site of implantation of the electrode array and stimulating configuration for cardiovascular stability-spinal cord epidural stimulation (CV-scES). **(A)** Sagittal and Axial MRI sections of the lumbosacral cord were used to calculate the cross-sectional area of the spinal cord and cerebrospinal fluid to create a 3-D reconstruction of the spinal cord at the lumbosacral enlargement. **(B)** Sagittal x-rays indicating the final placement of the electrode array. **(C)** 3-D reconstruction of the electrode array relative to the lumbosacral enlargement. **(D)** Graphical representation of electrode configuration for CV-scES, configuration uses two programs (P1 and P2) running concurrently. Cathodes are represented in black and anodes in gray. Each program runs with an independent stimulating amplitude, pulse width, and frequency.

One individual (female, 26.9 years old, 3.1 years from initial SCI) with chronic cervical motor-complete (C4, AIS grade A) SCI who presented with significant cardiovascular dysfunction was implanted with a 16-electrode array epidural stimulator (RestoreAdvanced, Medtronic) on the dura between spinal segments L1–S1 at the T11-L1 vertebral level (Figure 1). Parameters for CV-scES, including electrode polarity, frequency, and pulse width (Figure 1), were optimized to maintain systolic blood pressure within a normative range of 110–120 mmHg (Figure 1) without eliciting lower extremity or core muscle activation (Figure 1). The participant was instructed to use this electrode configuration at home throughout 97-sessions. Hemodynamic and immunological data were obtained before (pre) and after (post) the CV-scES intervention *without* active use of the stimulator during data collection. Data for the pre-CV-scES intervention were obtained before surgery. Data for the post-CV-scES intervention were obtained at least 24-h after the last stimulation session.

### Medical History

The individual sustained a cervical SCI from a motor vehicle accident at 24 years of age. Hardware was implanted to fuse the C6 vertebrae after initial SCI and was later revised due to the hardware-related infection. Medication history before study enrollment included Gabapentin for neurological pain, Oxybutynin Chloride for neurogenic bladder, and Methenamine Hippurate for prophylactic urinary tract infection prevention; the individual was gradually weaned from Gabapentin before surgery with guidance from her physiatrist but was allowed to continue the use of Oxybutynin Chloride and Methenamine Hippurate. In her medical history, the individual reported antibiotic treatment for recurrent urinary tract infections, that occurred at an average rate of ten times per year. There was no history of cardiovascular or pulmonary disease unrelated to SCI, but she reported persistent low blood pressure, episodic orthostatic hypotension, and decreased respiratory functional performance. Three months post-surgery and after only 12 CV-scES training sessions, the individual was struck by an oncoming car and sustained an intertrochanteric fracture of the left femur; after which, participation in the research study was placed on medical hold. The femur fracture was surgically repaired with an intramedullary Gamma nail and hip screw. The individual was an inpatient for a total of 14 days and was discharged to a rehabilitation hospital with recommendations to add Eliquis to her daily medications after developing a pulmonary embolism after surgery. At the rehabilitation hospital, the individual participated in standard physical and occupational therapy, including bed mobility, upright sitting, and upper extremity exercises. After 15 days as an inpatient at the rehabilitation hospital, the individual expressed interest in continuing in the research study. After medical clearance by her physiatrist, the study-physician, and the Data Safety Monitoring Board, the individual resumed study participation 53-days after the accident occurred.

### Hemodynamic Data Acquisition

Hemodynamic assessments were recorded in the morning in a quiet, temperature-controlled room. The participant was instructed to avoid consuming caffeine, alcohol, and nicotine products for at least 12-h and asked to empty her bladder upon arrival. Instrumentation was applied supine and included a three-lead ECG (Finapres Medical Systems, Amsterdam, Netherlands); beat-to-beat blood pressure recorded from the index finger, middle finger, or thumb using photoplethysmography (Finapres Medical Systems, Amsterdam, Netherlands); and transcranial Doppler (TCD) ultrasound of the middle cerebral artery (Terumo Cardiovascular Systems 1311 Valencia Avenue Tustin, CA 92780-6447, USA). Brachial blood pressure was recorded (GE Healthcare, Milwaukee, WI, USA) to calibrate beat-to-beat blood pressure offline (Bos et al., 1996).

The middle cerebral artery was insonated through the left temporal window at a frequency of 2.0 MHz. The middle cerebral artery was identified by the target depth (45–55 mm), sound and direction of flow (i.e., towards the probe), the characteristic spectral waveform, and relatively faster flow velocity compared with surrounding cerebral vessels. Once the middle cerebral artery was visualized, probe placement was secured for the duration of testing using a head-harness. Data output from the TCD was monitored in real-time and included systolic flow velocity, diastolic flow velocity, and mean flow velocities, recorded in centimeters per second (cm/s); quality control for TCD recordings was implemented by identifying a clear waveform, characterized by an upstroke to systolic peak and gradual decline to diastolic trough.

Acute orthostatic stress was induced with a passive, head-up tilt to 70° using a Hi-Lo Tilt Table (Hausman Industries, Inc., Northvale, NJ, USA). The individual was strapped to the bed at the tibial tuberosity, iliac crest, and below the axilla with her feet against the footplate. The individual rested quietly for 5 min in the supine position and then the bed was tilted to 70°, during which time blood pressure, ECG, and cerebral blood flow velocity were recorded continuously. The individual remained in the head-up tilt position for 30-min or until symptom-limited, in which case the test was immediately terminated. The head-up tilt test was performed before implantation and after 97-sessions of the CV-scES intervention; both assessments were performed without active CV-scES.

### Hemodynamic Data analysis

Digitized blood pressure and heart rate signals were analyzed with a custom program (MATLAB, The Mathworks, Natick, MA, USA) that performed R-peak detection of ECG, and peak and trough detection of the blood pressure waveform. Systemic mean arterial pressure (MAP) was calculated from the finger waveform as [systolic blood pressure + (2*diastolic blood pressure)]/3. Flow velocities of the middle cerebral artery were analyzed offline using a custom program written with LabVIEW graphical software (National Instruments, Austin TX, USA). Cerebral mean arterial pressure (cMAP) was estimated as the difference between systemic MAP and the hydrostatic pressure gradient calculated between the heart and the head (i.e., 17.8 mmHg). Mean cerebral blood flow velocity (mCBFv) was calculated from the integrated TCD signal over each cardiac cycle. The cerebrovascular conductance index was calculated as mCBFv/cMAP; cerebral autoregulatory function was estimated as the correlation coefficient between mCBFv and cMAP.

### Immunological Acquisition and Analysis

Blood for gene expression analysis was drawn from a butterfly catheter inserted into an antecubital vein pre and post-CV-scES intervention. Up to 8 ml of whole blood were collected in PAXgene tubes and stored at −20°C until analysis. Total RNA was isolated by QIAcube, using the manufacturer’s protocol (Qiagen, Venlo, Netherlands). RNA quality was determined on the Agilent Bioanalyzer, mRNA-Seq libraries were prepared (Illumina TruSeq Stranded Total RNA with RiboZero Globin, Catalog #20020612) and 100 bp paired-end reads were collected on the Illumina HiSeq 2500 platform (Yale Center for Genome Analysis). Using Partek Genomics Flow software (St. Louis, MO, USA), trimmed reads were aligned using STAR to the human genome (hg38 genome assembly), filtered for expression >50, normalized using the Trimmed Mean of *M*-values (TMM) method using the edgeR package embedded in Partek Genomics, and log2 transformed. For an initial comparison with a sample size of convenience, mRNA-Seq libraries were also prepared from three able-bodied individuals (*N* = 2 males, one female; ages 63, 64, and 66, respectively). Transcripts that were differentially expressed according to the between or within-participant(s) comparisons described below were identified with a fold change greater than 1.5, using the step-up method of the Benjamini–Hochberg method to correct *p* values with a false discovery rate (FDR) = 0.05 (Partek Genomics Flow). Principal components analysis (PCA) was performed using default parameters for the determination of the component number, with all components contributing equally (Partek Genomics Flow). Component loadings for each comparison examined can be found in Supplementary Table 1. For functional analysis of differentially expressed genes, if multiple transcripts for the same gene symbol were differentially expressed, then the transcript with the lowest *p*-value for that gene symbol by were included for further analyses. Lists of differentially expressed genes for each comparison examined can be found in Supplementary Table 1.

For functional analysis of differentially expressed genes, if multiple transcripts or RNA sequences for the same gene were differentially expressed, then the transcript with the lowest significant *p*-value was selected for directionality and further analyses.

Individual differentially expressed genes of interest are described below. Functional analysis of all differentially expressed genes (using unique gene symbols) in a particular comparison was performed using Enrichr (Chen et al., 2013), as we have done previously (Herman et al., 2018) to compare samples from able-bodied with samples from individuals with SCI (obtained before scES intervention), and within this research study to compare samples obtained pre and post-CV-scES intervention. Enrichr is an online platform that hosts independent gene-set libraries, or bioinformatics tools, that can be used to analyze functions of differentially expressed genes based on published scientific literature and publicly available data sets (Chen et al., 2013). Broadly speaking, the tools are divided into categories that characterize the function(s) of genes queried according to their known roles in signaling pathways, gene ontologies (GO; Ashburner et al., 2000; Carbon et al., 2019), cell types, or other functions such as transcription factors. While each bioinformatics tool may vary concerning specific naming of individual categories of enriched genes, biological meaning can be inferred when there is a thematic agreement across multiple independent platforms.

## Results

### Hemodynamic Data

**Figure 2:**
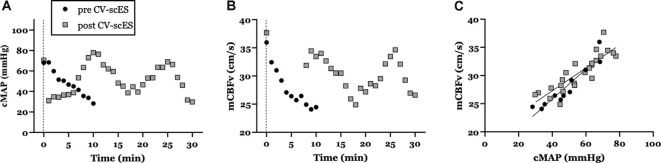
Mean arterial pressure (MAP) and cerebral blood flow during the orthostatic challenge are improved with CV-scES. **(A)** Cerebral mean arterial pressure (cMAP), **(B)** mean cerebral blood flow (mCBFv) during the head-up tilt maneuver, and **(C)** relationship between cMAP and mCBFv pre CV-scES (black circles) and post CV-scES (gray squares). Note that the participant was only able to tolerate 10-min in the 70° head-up tilt position pre-CV-scES, but was able to tolerate 30-min post-CV-scES.

Application of 97-sessions of CV-scES targeted to increase systolic blood pressure resulted in substantially improved cardiovascular and cerebrovascular responses during the head-up tilt maneuver. Orthostatic tolerance to 70° head-up tilt improved dramatically: from 10-min pre-CV-scES intervention to the full 30-min post-CV-scES intervention. Compared to pre-CV-scES intervention, improved orthostatic tolerance during head-up tilt post-CV-scES intervention was associated with increased cMAP (from 48.1 ± 13.3 to 51.8 ± 14.6 mmHg, respectively; Figure 2A) and increased mCBFv (from 28.0 ± 3.8 to 30.5 ± 3.2 cm/s, respectively; Figure 2B). Indeed, the slope of the relationship between cMAP and mCBFv pre CV-scES intervention (0.32 cm*s^−1^*mmHg^−1^) was decreased compared with post CV-scES intervention (0.16 cm*s^−1^*mmHg^−1^; Figure 2C). Changes to blood pressure while resting were minimal (pre-CV-scES: 112 ± 2.3/73 ± 2.3 mmHg; post-CV-scES: 114 ± 2.6/75 ± 1.6 mmHg).

### Immunological Results and Discussion

**Figure 3:**
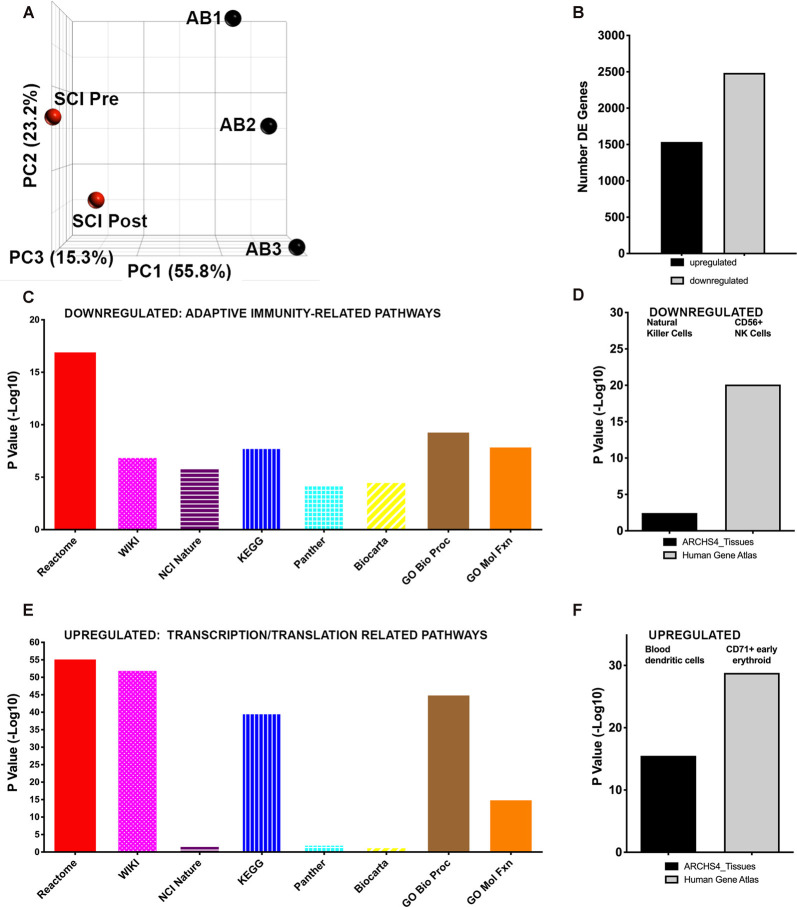
Whole blood gene expression is profoundly changed in an individual with spinal cord injury (SCI) compared with able-bodied (AB) individuals.** (A)** Principal component analysis (PCA) shows patterns of gene expression. Black symbols represent data obtained from three AB individuals, each sampled once. Red symbols represent data obtained from the individual with chronic SCI, pre and post CVscES intervention. PCA gene expression in the individual with SCI is similar pre- and post-intervention along the *Y*-axis (PC2) and *X*-axis (PC1), but is different on the *Z*-axis (PC3) axis. **(B)** The number of differentially expressed genes (up- or down-regulated) is shown. **(C)** Downregulated genes: multiple bioinformatics platforms independently identified pathways enriched following usual care in the participant with SCI, which are related to adaptive immunity. **(D)** Two cell type bioinformatics platforms identified natural killer (NK) cell genes enriched among downregulated genes. **(E)** Upregulated genes: multiple bioinformatics platforms independently identified pathways enriched following usual care in the participant with SCI that are related to viral and eukaryotic transcription and translation. **(F)** Two cell type bioinformatics platforms identified dendritic cells and CD71+ early erythroid progenitor cells genes enriched among upregulated genes.

**Figure 4:**
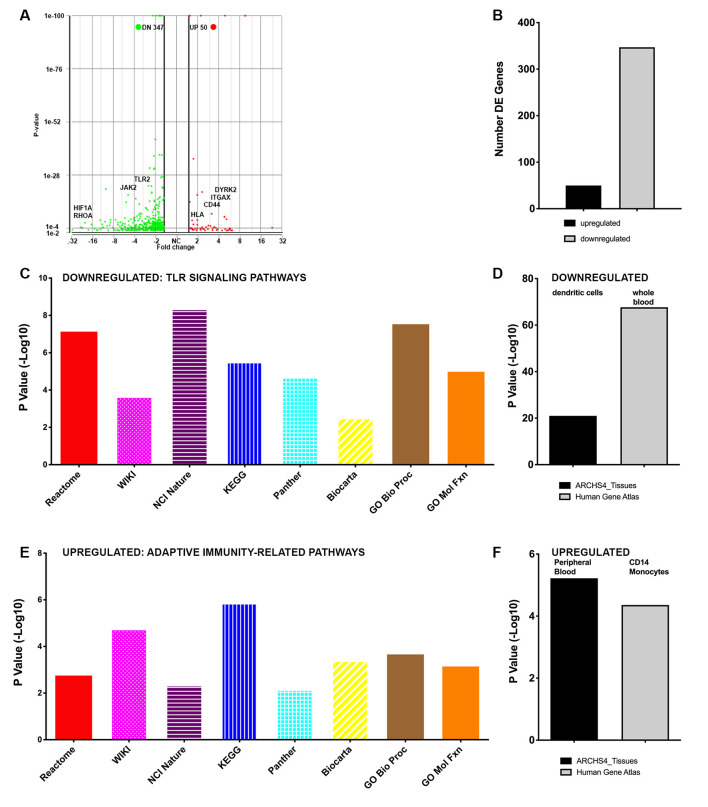
Whole blood gene expression is profoundly changed within an individual with SCI after 80 sessions of CV scES. **(A)** The volcano plot shows several differentially expressed genes within the participant following 97-sessions of CV-scES. Downregulated genes (green) include pro-inflammatory genes of interest, such as JAK2, RHOA, and toll-like receptors (TLRs). Upregulated genes (red) include genes of interest related to adaptive immunity, e.g., CD44 and HLA related molecules (MHCI and MHCII). **(B)** The numbers of differentially expressed genes are shown. **(C)** Downregulated genes: multiple bioinformatics platforms independently identified pathways related to pro-inflammatory TLR signaling. **(D)** Cell type bioinformatics platforms identified whole blood and dendritic cells enriched among downregulated genes. **(E)** Upregulated genes: multiple bioinformatics platforms independently identified pathways related to adaptive immunity. **(F)** Two cell type bioinformatics platforms identified peripheral blood and CD14+ monocytes enriched among upregulated genes.

We first analyzed major variations in whole blood gene expression in blood obtained pre CV-scES intervention from the individual with chronic SCI compared with able-bodied (AB) individuals. This is illustrated by principal component analysis (Figure 3A, Supplementary Table 1), where the first component explained 61.88% of the total variation in gene expression. Compared to uninjured individuals, in the sample obtained from the individual with SCI before CV-scES intervention, there were a total of 4019 differentially expressed unique genes (FDR = 0.05). Of these, there were 1,535 up- and 2,485 down-regulated genes in the individual with chronic SCI compared with able-bodied (AB) data (Figure 3B). We used multiple independent bioinformatics platforms to identify common functional themes among pathways enriched in the genes differentially expressed in the individual with SCI after usual care compared to the AB data. Interestingly, multiple bioinformatics platforms identified downregulated pathways that were related to adaptive immunity, antigen processing, and presentation, which is consistent with a clinical phenotype of immunosuppression (Figure 3C). By platform, these were: REACTOME = adaptive immune system (R-HSA-1280218), WIKI = B cell receptor signaling pathway (WP23), NCI-Nature = IL2-mediated signaling events (a2a1883c-6193-11e5-8ac5-06603eb7f303), KEGG = antigen processing and presentation, Panther = T cell activation (P00053), BioCarta = role of MEFD in T-cell Apoptosis. The Gene Ontology (GO) platform (Ashburner et al., 2000; Carbon et al., 2019), which categorizes known functions of biological molecules by drawing on data from many species, was used to analyze the enrichment of differentially expressed genes using the Biological Process ontology, which describes how genes contribute to multiple biological processes (geneontology.org). highlighted regulation of gene expression related to T cell receptor signaling (GO: 0050852), as enriched among downregulated transcripts. GO analysis using the Molecular Function platform, which describes gene-related activities at the molecular level, highlighted MHCII protein complex binding (GO: 0023026), which is part of the antigen presentation molecular machinery, as enriched among downregulated transcripts. Interestingly, cellular level analysis using the ARCHS4 tissues platform also identified the downregulation of genes enriched in NK Cells (2.45-Log10 *p*-value) of the innate immune system, which are critical for maintaining anti-viral immunity (Figure 3D). The Human Gene Atlas independently identified downregulated genes as highly enriched for CD56+ NK Cells transcripts (20.10, −Log10 *p*-value; Figure 3D). Multiple bioinformatics platforms identified pathways enriched among upregulated genes that were related to both eukaryote and viral transcription and translation, as well as cell cycle genes and pro-inflammatory genes (Figure 3E). By platform, these included: REACTOME = viral mRNA Translation (R-HSA-192823), WIKI = cytoplasmic ribosomal proteins (WP477), NCI-Nature = signaling events mediated by focal adhesion kinase (8fb80085-6195-11e5-8ac5-06603eb7f303), KEGG = Ribosome, Panther = *De novo* pyrimidine deoxynucleotide synthesis (P02739), BioCarta = Cyclin E destruction pathway. Gene Ontology (GO) analysis using the Biological Process platform highlighted viral gene expression (GO: 00190980), as enriched among downregulated transcripts. GO analysis using the Molecular Function platform highlighted RNA binding (GO: 0003723), which part of the antigen presentation machinery, as enriched among downregulated transcripts. Cellular level analysis using the ARCHS4 tissues platform also identified the upregulation of genes enriched in blood dendritic cells (Figure 3F), which are highly potent antigen-presenting cells that link the innate and adaptive immune system. The Human Gene Atlas identified upregulated genes as highly enriched for CD71+ (transferrin receptor) early erythroid progenitors (Figure 4F) and CD105+ endothelial cells (5.12 −Log10 *p*-value), among others.

We next analyzed changes in gene expression in the participant with SCI in response to CV-scES. After 97-sessions daily CV-scES, interestingly, 397 differentially expressed genes were observed compared with pre-CV-scES intervention, with 50 upregulated and 347 downregulated unique gene symbols (Figures 4A,B). At the gene level, several key pro-inflammatory genes of interest were downregulated, such as members of the MAP kinase family, TLRs-1, 2, and 4, and LY96, which interacts with TLR4 (Figure 4A). As with the analysis above, we used several independent bioinformatics platforms to identify pathways of genes that were differentially expressed post-CV-scES intervention compared with pre-CV-scES intervention within the individual with SCI. Interestingly, multiple bioinformatics platforms identified downregulated pathways related to the pro-inflammatory TLR signaling, which is critical for pathogen sensing by the innate immune system (Figure 4C). By platform, these were: REACTOME = Activated TLR signaling (R-HSA-166054), WIKI = SCI pathway (WP2431), NCI-Nature = endogenous TLR signaling (d8777e16-6191-11e5-8ac5-06603eb7f303), KEGG = tuberculosis, Panther = Toll receptor signaling pathway (P00054), BioCarta = Toll-like receptor pathway. Gene Ontology (GO) analysis using the Biological Process platform highlighted TLR signaling (GO: 0002224), as enriched among downregulated transcripts. GO analysis using the Molecular Function platform also highlighted TLR binding (GO: 003525), as enriched among downregulated transcripts. Also, cellular components analysis using the ARCHS4 tissues platform identified the downregulation of genes enriched in the potent antigen-presenting cells, dendritic cells (21.00 −Log10 *p*-value;). The Human Gene Atlas identified downregulated genes as highly enriched for whole blood transcripts (67.7, −Log10 *p*-value; Figure 4D). Multiple bioinformatics platforms identified pathways enriched among upregulated genes that were related to adaptive immunity, including antigen processing and presentation (Figure 4E). By platform, these included: REACTOME = antigen presentation: folding, assembly and peptide loading of class I MHC (R-HSA-983170), WIKI = ebola virus pathway on the host (WP4217) which included the class I and II MHC molecules HLA-A, HLA-B and HLA-DR, NCI-Nature = integrin signaling (5d4f90b6-6188-11e5-8ac5-06603eb7f303), KEGG = antigen processing and presentation, Panther = integrin signaling pathway (P00034), BioCarta = antigen processing and presentation. GO analysis using the Biological Process platform highlighted the upregulation of gene expression related to antigen processing and presentation *via* MHCI (GO:0002480). GO analysis using the Molecular Function platform also highlighted antigen presentation *via* MHCII (GO: 0023026), as enriched among upregulated transcripts. Cellular components analysis using the ARCHS4 tissues platform also identified the upregulation of genes enriched in peripheral blood (Figure 4F), among others. The Human Gene Atlas identified upregulated genes as highly enriched in CD14+ monocytes, among others (Figure 4F). GO analysis using the Biological Process platform highlighted regulation of gene expression and cellular response to lipopolysaccharide (GO: 0071222), a major ligand for TLR4, as enriched among downregulated transcripts.

### Self-reported Results

Following 97-sessions of the CV-scES intervention, the participant reported that her blood pressure no longer fell precipitously during daily activities and, as a result, her health and feelings of vitality improved. Because her blood pressure is stable and closer to a normotensive range, she reports that she has gained confidence, independence, and autonomy, allowing her to: participate in in social gatherings with friends and dating; return to college to finish a degree she started pre-injury; and feel a stronger connection within the community. Simple activities of daily life that previously required assistance have become a part of her daily routine, including brushing her teeth or her hair, washing alone in the shower, and applying her makeup without having to take breaks. Importantly, the participant was an avid singer before her accident, and she reports that she has regained the cardiovascular strength and stamina to allow her to return to singing loudly and to carry notes for an extended period. Finally, the participant reports significantly improved health such that she no longer feels “sick” and reported significantly reduced hospital and doctor’s office visits for upper respiratory and urinary tract infections. Before the implant, the participant reported an annual incidence of ten urinary tract infections. In the 18 months, the individual has been using the stimulator, she has been diagnosed with and treated for three urinary tract infections without a change to her bladder maintenance program.

## Discussion

Findings of the current case report demonstrate that prolonged, targeted CV-scES intervention leads to improved blood pressure regulation, increased orthostatic tolerance, and improved cMAP and mCBFv responses during a head-up tilt maneuver even when assessed without stimulation during testing. There was also a reduction in the slope of the relationship cMAP and mCBFv during head-up tilt, suggesting improved cerebral autoregulation post-CV-scES intervention. Further, after the CV-scES intervention, genes related to adaptive immunity were upregulated, while genes related to TLR signaling were downregulated across multiple bioinformatics platforms, which would be consistent with improved immunity and reduced systemic inflammation, respectively. These improvements that persist post-CV-scES intervention without active stimulation suggest daily use of targeted CV-scES has a beneficial impact on multiple organ systems that are regulated by the ANS.

Spinal cord epidural stimulation has a long history for use in treating pain beginning in the 1960s (Shealy et al., 1967), which was FDA-approved in 1989, and has been shown to improve microvascular circulation in peripheral vascular disease (Jacobs et al., 1988). Epidural stimulation of the lumbosacral motor circuitry has been studied experimentally in animals (Gerasimenko, 2002) as well as in humans for spasticity and to understand network circuitry for locomotion following SCI (Dimitrijevic et al., 1998; Hargens and Richardson, 2009; Harkema et al., 2016; Grahn et al., 2017; Rejc et al., 2017a,b; Angeli et al., 2018). This approach takes advantage of sophisticated spinal networks below the level of injury to unexpectedly reactivate spared pathways, restoring supraspinal connectivity and resulting in functional recovery in those initially diagnosed clinically as motor complete (Rejc et al., 2017b, Calvert et al., 2019).

Investigators recently discovered that specific cardiovascular-targeted scES configurations (CV-scES, L1-S4/5 spinal cord), that did not elicit motor activity in individuals with SCI who report cardiovascular dysfunction, could increase and stabilize orthostatic blood pressure during the orthostatic challenge (Harkema et al., 2018; West et al., 2018; Darrow et al., 2019), and mitigate symptoms reporting associated with orthostatic intolerance. An interesting and novel finding from these studies was that orthostatic blood pressure was maintained after 80-sessions of CV-scES in 4 participants with chronic severe motor complete SCI, even with the stimulator turned off, which suggests that targeted CV-scES may facilitate adaptive neuroplasticity to restore endogenous ANS function (Harkema et al., 2018). Two recent case studies confirm the cardiovascular autonomic benefits following a single session of targeted scES in response to an orthostatic challenge (West et al., 2018; Darrow et al., 2019). Furthermore, improvement in cerebral blood flow velocity and cognitive function was reported during a 70° head-up tilt maneuver in a female participant with cardiovascular deficits in response to epidural stimulation of the thoracolumbar cord.

Profiling of whole blood gene expression in this individual pre-CV-scES intervention demonstrated molecular signatures consistent with reduced adaptive immune responses, as we saw previously in a larger cohort of individuals with chronic SCI (*N* = 31) vs. able-bodied persons (*N* = 26; Herman et al., 2018). For example, multiple pathway analysis platforms identified genes related to T and B cell receptor signaling, required for antigen processing and presentation, as downregulated compared with able-bodied persons. Two independent cell type platforms identified genes enriched in NK cells, which are critical for killing virally infected cells, as downregulated, as well as general PBMC genes and other T and B cell genes. These findings of downregulated genes are consistent with a clinical phenotype of increased infection susceptibility. Pre CV-scES intervention, genes that were upregulated compared with able-bodied persons were related to eukaryotic and viral transcription and translation. In contrast, within the individual with SCI post-CV-scES intervention, genes related to adaptive immunity were upregulated across multiple bioinformatics platforms, which would be consistent with improved immunity. Interestingly, after CV-scES, downregulated genes were related to TLR signaling, a pathway that is critical for pathogen sensing by the innate immune system and promotes inflammation, which we previously identified as upregulated in individuals with chronic SCI (Herman et al., 2018). This would be consistent with reduced systemic inflammation.

There are several limitations to this proof-of-concept case report, including this is a report of effects in a single individual and therefore need to be validated in studies with a larger number of patients, the longer-term duration of effects is unknown, the optimal stimulation parameters for immune-related responses is unknown, and there may be unknown potential confounders that influence the observed effects. While defining the mechanisms that underlie this immune response to targeted epidural stimulation is beyond the scope of this case-report, this should be explored in future studies. Several pre-clinical studies in SCI have shown that an intact sympathetic nervous system is critical for proper immune function (Schwab et al., 2014). Furthermore, pre-clinical studies and clinical trials have shown that the parasympathetic nervous system is also critical for regulating inflammation and other aspects of immunity (Koopman et al., 2016; Pavlov and Tracey, 2017). Similarly, identifying the ANS changes that are responsible for the improved systemic and cerebral hemodynamics following prolonged application of CV-scES is essential and may impact (directly and indirectly) the observed effects on the immune system. As this is a single case study, additional efforts are ongoing to examine whether this effect is consistent in other participants receiving CV-scES and whether this effect is specific to a CV-targeted configuration. Despite these limitations, taken together, these data support the hypothesis that targeted CV-scES parameters that modulate blood pressure may elicit other improvements in ANS-regulated functions, including those that promote beneficial changes in the immune system.

## Data Availability Statement

The raw data supporting the conclusions of this article will be made available by the authors, without undue reservation.

## Ethics Statement

The studies involving human participants were reviewed and approved by the local IRB of University of Louisville (NCT 03364660); the portion of the work performed at Northwell Health was reviewed by the local IRB and deemed exempt. The patients/participants provided their written informed consent to participate in this study. Written informed consent was obtained from the individual for the publication of any potentially identifiable images or data included in this article.

## Author Contributions

OB, JW, BD, SW, AO, CA, AA, and SH collected and analyzed data. OB, JW, BD, AO, and CA wrote the manuscript. SH and CA designed the clinical trial and the project. All authors contributed to the article and approved the submitted version.

## Conflict of Interest

The authors declare that this study received funding from Medtronic Plc in the form of implantable devices. The funder was not involved in the study design, collection, analysis, interpretation of data, the writing of this article, or the decision to submit it for publication.

## Supplementary Material

SUPPLEMENTARY TABLE 1 Lists of differentially expressed genes. Gene symbols of differentially expressed genes (with redundant gene symbols removed as described in “Materials and Methods” section) are provided for each comparison as described in the text. Worksheets “SCI Pre CVscES vs. AB DE genes” lists the genes differentially expressed in the participant with SCI before CV-scES compared to able-bodied persons, “PCA Component loadings” shows the transcript loading components for each of five PCs in the PCA comparing the participant with SCI to the able-bodied persons, “SCI Post vs. Pre CVscES DE genes lists the genes differentially expressed in the participant with SCI after the 97 sessions compared to before the CV-scES.
